# Supplementary material for: Role of Magnesium in Ultra-Low-Radioactive Titanium Production for Future Direct Dark Matter Search Detectors
Source: Materials (Basel). 2022 Dec 12;15(24):8872. doi: 10.3390/ma15248872 (PMC9786686; doi:10.3390/ma15248872)
Supplement: Supplementary file 1 [file materials-15-08872-s001.zip › materials-2073040-supplementary.pdf]

materials

MDPI

Supplementary

Magnesium Role in Ultra-Low-Radioactive Titanium Production for Future Direct Dark Matter Search Detectors

Marina Zykova, Elena Voronina, Alexander Chepurnov, Dmitry Rymkevich, Aleksey Tankeev, Sergey Vlasov, Alexander Chub and Igor Avetissov

Table S1. The ICP-MS results of Mg purified by vacuum distillation and electrolysis.

	Mg by Electrolysis	Mg by Vacuum Distillation		
		Mg-S-Top	Mg-S-Bot	Mg-S-Cube
Element	Wt%	Wt%	Wt%	Wt%
Li	$4.01 \cdot 10^{-5}$	$7.34 \cdot 10^{-6}$	$1.40 \cdot 10^{-6}$	$1.10 \cdot 10^{-6}$
Be	$< 3.34 \cdot 10^{-7}$	$1.10 \cdot 10^{-6}$	$< 7.08 \cdot 10^{-7}$	$< 8.31 \cdot 10^{-7}$
B	$< 1.39 \cdot 10^{-5}$	$< 1.68 \cdot 10^{-5}$	$4.40 \cdot 10^{-5}$	$1.61 \cdot 10^{-4}$
Na	matrix	$3.91 \cdot 10^{-4}$	$4.82 \cdot 10^{-4}$	$3.04 \cdot 10^{-4}$
Mg	matrix	matrix	matrix	matrix
Al	$3.95 \cdot 10^{-4}$	$8.63 \cdot 10^{-5}$	$9.97 \cdot 10^{-5}$	$1.95 \cdot 10^{-3}$
Si	$9.42 \cdot 10^{-4}$	$5.73 \cdot 10^{-4}$	$3.95 \cdot 10^{-4}$	$8.20 \cdot 10^{-4}$
K	matrix	$3.17 \cdot 10^{-3}$	$3.36 \cdot 10^{-3}$	$3.09 \cdot 10^{-3}$
Ca	$2.55 \cdot 10^{-1}$	$9.74 \cdot 10^{-4}$	$5.60 \cdot 10^{-4}$	$8.13 \cdot 10^{-4}$
Sc	$3.12 \cdot 10^{-7}$	$< 3.04 \cdot 10^{-5}$	$< 3.16 \cdot 10^{-5}$	$< 3.71 \cdot 10^{-5}$
Ti	$1.28 \cdot 10^{-4}$	$1.39 \cdot 10^{-4}$	$2.03 \cdot 10^{-4}$	$1.25 \cdot 10^{-2}$
V	$2.12 \cdot 10^{-5}$	$7.84 \cdot 10^{-7}$	$2.46 \cdot 10^{-7}$	$2.11 \cdot 10^{-6}$
Cr	$1.52 \cdot 10^{-5}$	$1.29 \cdot 10^{-5}$	$2.04 \cdot 10^{-5}$	$3.18 \cdot 10^{-3}$
Mn	$1.60 \cdot 10^{-4}$	$1.27 \cdot 10^{-5}$	$1.79 \cdot 10^{-5}$	$4.67 \cdot 10^{-4}$
Fe	$3.03 \cdot 10^{-3}$	$1.39 \cdot 10^{-4}$	$2.24 \cdot 10^{-4}$	$1.04 \cdot 10^{-3}$
Co	$7.72 \cdot 10^{-7}$	$4.45 \cdot 10^{-7}$	$6.87 \cdot 10^{-7}$	$1.72 \cdot 10^{-6}$
Ni	$1.54 \cdot 10^{-5}$	$6.83 \cdot 10^{-5}$	$1.47 \cdot 10^{-5}$	$1.62 \cdot 10^{-3}$
Cu	$2.67 \cdot 10^{-5}$	$3.46 \cdot 10^{-6}$	$1.00 \cdot 10^{-6}$	$1.10 \cdot 10^{-4}$
Zn	$< 6.01 \cdot 10^{-6}$	$4.79 \cdot 10^{-3}$	$1.59 \cdot 10^{-2}$	$4.32 \cdot 10^{-3}$
Ga	$< 4.46 \cdot 10^{-6}$	$1.98 \cdot 10^{-6}$	$2.89 \cdot 10^{-6}$	$2.95 \cdot 10^{-6}$
Ge	$4.72 \cdot 10^{-6}$	$5.53 \cdot 10^{-6}$	$3.92 \cdot 10^{-6}$	$3.31 \cdot 10^{-6}$
As	$2.34 \cdot 10^{-5}$	$1.15 \cdot 10^{-5}$	$3.65 \cdot 10^{-6}$	$7.42 \cdot 10^{-6}$
Se	$5.27 \cdot 10^{-5}$	$9.19 \cdot 10^{-5}$	$< 4.96 \cdot 10^{-5}$	$9.16 \cdot 10^{-5}$
Rb	$4.16 \cdot 10^{-2}$	$< 1.85 \cdot 10^{-6}$	$< 1.92 \cdot 10^{-6}$	$2.59 \cdot 10^{-6}$
Sr	$7.83 \cdot 10^{-4}$	$1.08 \cdot 10^{-6}$	$1.08 \cdot 10^{-6}$	$1.69 \cdot 10^{-6}$
Y	$4.26 \cdot 10^{-5}$	$2.58 \cdot 10^{-7}$	$2.14 \cdot 10^{-7}$	$1.57 \cdot 10^{-7}$
Zr	$9.52 \cdot 10^{-6}$	$9.14 \cdot 10^{-7}$	$1.40 \cdot 10^{-6}$	$1.77 \cdot 10^{-6}$
Nb	$1.13 \cdot 10^{-6}$	$4.86 \cdot 10^{-7}$	$1.18 \cdot 10^{-7}$	$6.15 \cdot 10^{-7}$
Mo	$2.87 \cdot 10^{-6}$	$< 1.57 \cdot 10^{-5}$	$< 1.63 \cdot 10^{-5}$	$< 1.92 \cdot 10^{-5}$
Ru	$4.25 \cdot 10^{-7}$	$< 2.04 \cdot 10^{-8}$	$< 2.12 \cdot 10^{-8}$	$< 2.49 \cdot 10^{-8}$
Rh	$8.83 \cdot 10^{-7}$	$< 3.41 \cdot 10^{-8}$	$< 3.54 \cdot 10^{-8}$	$9.83 \cdot 10^{-8}$
Pd	$< 8.43 \cdot 10^{-8}$	$< 1.70 \cdot 10^{-8}$	$< 1.77 \cdot 10^{-8}$	$6.86 \cdot 10^{-8}$
Ag	$9.28 \cdot 10^{-8}$	$< 6.68 \cdot 10^{-8}$	$< 6.95 \cdot 10^{-8}$	$< 8.16 \cdot 10^{-8}$
Cd	$3.65 \cdot 10^{-7}$	$2.73 \cdot 10^{-6}$	$7.10 \cdot 10^{-6}$	$1.74 \cdot 10^{-6}$
In	$< 1.45 \cdot 10^{-7}$	$< 1.93 \cdot 10^{-6}$	$< 2.01 \cdot 10^{-6}$	$< 2.36 \cdot 10^{-6}$
Sn	$< 1.57 \cdot 10^{-6}$	$2.75 \cdot 10^{-7}$	$< 2.80 \cdot 10^{-7}$	$6.41 \cdot 10^{-6}$

Sb	$1.04\cdot10^{-6}$	$< 2.16\cdot10^{-7}$	$4.02\cdot10^{-7}$	$< 2.63\cdot10^{-7}$
Te	$< 1.28\cdot10^{-5}$	$< 3.96\cdot10^{-6}$	$< 4.11\cdot10^{-6}$	$< 4.83\cdot10^{-6}$
Cs	$1.77\cdot10^{-5}$	$4.31\cdot10^{-7}$	$< 2.20\cdot10^{-7}$	$< 2.58\cdot10^{-7}$
Ba	$7.73\cdot10^{-4}$	$1.70\cdot10^{-6}$	$2.65\cdot10^{-6}$	$3.26\cdot10^{-6}$
La	$5.50\cdot10^{-5}$	$5.11\cdot10^{-8}$	$< 2.04\cdot10^{-8}$	$2.48\cdot10^{-7}$
Ce	$1.85\cdot10^{-4}$	$< 4.68\cdot10^{-7}$	$< 4.86\cdot10^{-7}$	$< 5.71\cdot10^{-7}$
Pr	$1.43\cdot10^{-4}$	$< 3.15\cdot10^{-8}$	$3.30\cdot10^{-8}$	$< 3.84\cdot10^{-8}$
Nd	$1.36\cdot10^{-5}$	$< 6.81\cdot10^{-9}$	$< 7.08\cdot10^{-9}$	$2.45\cdot10^{-7}$
Sm	$2.46\cdot10^{-6}$	$< 6.81\cdot10^{-9}$	$< 7.08\cdot10^{-9}$	$< 8.31\cdot10^{-9}$
Eu	$9.56\cdot10^{-7}$	$< 6.81\cdot10^{-9}$	$< 7.08\cdot10^{-9}$	$< 8.31\cdot10^{-9}$
Gd	$2.51\cdot10^{-6}$	$< 1.50\cdot10^{-7}$	$< 1.56\cdot10^{-7}$	$< 1.83\cdot10^{-7}$
Tb	$6.32\cdot10^{-7}$	$< 6.20\cdot10^{-7}$	$< 6.44\cdot10^{-7}$	$< 7.56\cdot10^{-7}$
Dy	$3.43\cdot10^{-6}$	$< 6.81\cdot10^{-9}$	$< 7.08\cdot10^{-9}$	$< 8.31\cdot10^{-9}$
Ho	$6.08\cdot10^{-7}$	$< 6.81\cdot10^{-9}$	$< 7.08\cdot10^{-9}$	$< 8.31\cdot10^{-9}$
Er	$1.76\cdot10^{-6}$	$< 6.81\cdot10^{-9}$	$< 7.08\cdot10^{-9}$	$< 8.31\cdot10^{-9}$
Tm	$2.50\cdot10^{-7}$	$< 6.81\cdot10^{-9}$	$< 7.08\cdot10^{-9}$	$< 8.31\cdot10^{-9}$
Yb	$2.35\cdot10^{-6}$	$< 6.81\cdot10^{-9}$	$< 7.08\cdot10^{-9}$	$< 8.31\cdot10^{-9}$
Lu	$7.83\cdot10^{-8}$	$< 1.75\cdot10^{-7}$	$< 1.82\cdot10^{-7}$	$< 2.14\cdot10^{-7}$
Hf	$< 9.73\cdot10^{-9}$	$< 6.81\cdot10^{-9}$	$< 7.08\cdot10^{-9}$	$< 8.31\cdot10^{-9}$
Ta	$8.56\cdot10^{-8}$	$< 6.81\cdot10^{-9}$	$1.30\cdot10^{-8}$	$1.52\cdot10^{-8}$
W	$< 6.63\cdot10^{-8}$	$< 3.26\cdot10^{-7}$	$< 3.39\cdot10^{-7}$	$< 3.98\cdot10^{-7}$
Re	$< 9.73\cdot10^{-9}$	$< 6.81\cdot10^{-9}$	$< 7.08\cdot10^{-9}$	$< 8.31\cdot10^{-9}$
Os	$< 9.73\cdot10^{-9}$	$< 6.81\cdot10^{-9}$	$< 7.08\cdot10^{-9}$	$< 8.31\cdot10^{-9}$
Ir	$< 9.73\cdot10^{-9}$	$< 6.81\cdot10^{-9}$	$< 7.08\cdot10^{-9}$	$< 8.31\cdot10^{-9}$
Pt	$< 2.39\cdot10^{-6}$	$< 4.78\cdot10^{-7}$	$< 4.97\cdot10^{-7}$	$< 5.83\cdot10^{-7}$
Au	$< 2.34\cdot10^{-7}$	$< 1.22\cdot10^{-7}$	$< 1.27\cdot10^{-7}$	$< 1.49\cdot10^{-7}$
Hg	$< 7.76\cdot10^{-7}$	$2.64\cdot10^{-4}$	$1.40\cdot10^{-3}$	$2.30\cdot10^{-4}$
Tl	$< 1.30\cdot10^{-7}$	$2.38\cdot10^{-8}$	$7.90\cdot10^{-8}$	$1.90\cdot10^{-7}$
Pb	$2.30\cdot10^{-6}$	$9.08\cdot10^{-8}$	$< 5.66\cdot10^{-8}$	$1.38\cdot10^{-6}$
Bi	$1.17\cdot10^{-4}$	$2.71\cdot10^{-7}$	$< 1.38\cdot10^{-7}$	$< 1.62\cdot10^{-7}$
Th	$2.82\cdot10^{-7}$	$< 2.21\cdot10^{-7}$	$< 2.29\cdot10^{-7}$	$4.80\cdot10^{-5}$
U	$2.96\cdot10^{-7}$	$9.65\cdot10^{-9}$	$< 8.91\cdot10^{-9}$	$< 1.05\cdot10^{-8}$

Table S2. The ICP-MS results of Mg production via electrolysis process and Mg samples obtained by the various processes in comparison with $MgCl_2$ outputs from Kroll process.

Sample ID	Th concentration, ppb		U concentration, ppb	
	Content	Standard Deviation	Content	Standard Deviation
Carnallite				
C1	6	2	0.3	0.04
C2	0.9	0.01	0.51	0.02
C3	0.8	0.01	0.007	0.002
Dehydrated carnallite				
DC1	1.1	0.1	0.61	0.03
DC2	1.1	0.1	0.39	0.01
DC3	1.6	0.1	0.78	0.05
Dehydrated carnallite after chlorination				
DCC1	3.0	0.5	1.2	0.1
DCC2	3.4	0.2	1.9	0.1
DCC3	3.2	0.1	1.8	0.1

Mg (N1) production by electrolysis				
Mg №242 30.12.14	487	7	0.65	0.93
Mg №224 15.07.2014	60	2	<0.001	-
Mg № 223 15.07.2014	102	1	15	2
Mg (N2) production by electrolysis				
Mg E-1	37	2	7.7	0.4
Mg E-2	40	3	8.5	0.8
Mg E-3	36	3	1.6	0.2
$MgCl_2$ (N1) after metallothermic process				
$MgCl_2$ 1 15.08.2014	0.06	0.01	21.6	0.2
$MgCl_2$ 2 15.08.2014	0.25	0.03	10.4	0.2
$MgCl_2$ 3 15.08.2014	<0.001	-	<0.001	-
$MgCl_2$ (N2) after metallothermic process				
$MgCl_2$-E-1	0.4	0.1	0.06	0.01
$MgCl_2$-E-2	7.5	0.7	0.04	0.01
$MgCl_2$-E-3	5.9	0.4	0.64	0.03
Mg obtained by Pidgeon process				
Mg-China	2.52	0.73	0.001	0.0005
